# Comorbidity in patients with chronic obstructive pulmonary disease in family practice: a cross sectional study

**DOI:** 10.1186/1471-2296-14-11

**Published:** 2013-01-16

**Authors:** Luis García-Olmos, Ángel Alberquilla, Victoria Ayala, Pilar García-Sagredo, Leticia Morales, Montserrat Carmona, María José de Tena-Dávila, Mario Pascual, Adolfo Muñoz, Carlos H Salvador, Jose L Monteagudo

**Affiliations:** 1 Multiprofessional Education Unit for Family and Community Care (South-east), Hacienda de Pavones, 271, Madrid, 28030, Spain; 2 Multiprofessional Education Unit for Family and Community Care (Centre), Alberto Palacios, 22, Madrid, 28021, Spain; 3 Medical Department, Praxair España, Orense, 11, Madrid, 28020, Spain; 4 Telemedicine and e-Health Unit, Carlos III Institute of Health, Avda. Monforte de Lemos, 5, Madrid, 28029, Spain; 5 Bioengineering and Telemedicine Unit, Puerta de Hierro University Teaching Hospital, Majadahonda, Madrid, Spain

**Keywords:** COPD, Comorbidity, Family practice, Epidemiology

## Abstract

**Background:**

Chronic obstructive pulmonary disease (COPD) is frequent and often coexists with other diseases. The aim of this study was to quantify the prevalence of COPD and related chronic comorbidity among patients aged over 40 years visiting family practices in an area of Madrid.

**Methods:**

An observational, descriptive, cross-sectional study was conducted in a health area of the Madrid Autonomous Region (*Comunidad Autónoma de Madrid*). The practice population totalled 198,670 persons attended by 129 Family Physicians (FPs), and the study population was made up of persons over the age of 40 years drawn from this practice population. Patients were deemed to have COPD if this diagnosis appeared on their clinical histories. Prevalence of COPD; prevalence of a further 25 chronic diseases in patients with COPD; and standardised prevalence ratios, were calculated.

**Results:**

Prevalence of COPD in family medicine was 3.2% (95% CI 3.0–3.3) overall, 5.3% among men and 1.4% among women; 90% of patients presented with comorbidity, with a mean of 4 ± 2.04 chronic diseases per patient, with the most prevalent related diseases being arterial hypertension (52%), disorders of lipid metabolism (34%), obesity (25%), diabetes (20%) and arrhythmia (15%). After controlling for age and sex, the observed prevalence of the following ten chronic diseases was higher than expected: heart failure; chronic liver disease; asthma; generalised artherosclerosis; osteoporosis; ischaemic heart disease; thyroid disease; anxiety/depression; arrhythmia; and obesity.

**Conclusions:**

Patients with COPD, who are frequent in family practice, have a complex profile and pose a clinical and organisational challenge to FPs.

## Background

Chronic obstructive pulmonary disease (COPD) is frequent, incapacitating at advanced stages, leads to the death of the patient and, by 2030, is expected to be the fourth leading cause of death and the seventh leading cause of loss of disability adjusted life years (DALYs) [1].

Until the year 2000, few epidemiological studies had addressed COPD, and those that were undertaken mostly targeted the population aged over 40 years. A meta-analysis of studies published up to 2004 estimated COPD prevalence among over 40-year-olds to be 10% [2]; however, heterogeneity in the definition of case makes difficult results comparison. Latest estimations in Spain, using the GOLD criteria, set COPD prevalence in 10.2%, with strong variation among different regions and an important fall regarding a previous study performed ten years earlier [3].

Comorbidity is present in one third of the adult population and its prevalence increases with age, eventually affecting 60% of the population aged 55 to 74 years [4]; moreover, it has been observed that certain chronic diseases tend to be present in clusters [5].

COPD often coexists with other diseases, and this related comorbidity is a key prognostic factor of the consequences of COPD. Some of these diseases occur regardless of COPD, some are related by virtue of having a common cause(s), and others share risk factors [6]. For instance, inflammation brought about by smoking can be a common route for a series of diseases, such as ischaemic heart disease, heart failure, osteoporosis, anaemia, cancer, depression and diabetes [7]. COPD associated comorbidity contributes to symptoms, determines the quality of life [8,9], increments healthcare costs [10] and increments the mortality of patients suffering it [11].

Clinical research targets isolated diseases. Chronic care is geared to managing individual diseases (disease management programmes), and the clinical practice guidelines that underpin these models likewise focus on isolated diseases [12]. Stratification of COPD patients takes into account the level of obstruction, but does not consider the burden of comorbidity, notwithstanding its implications.

Research into comorbidity is scant, and relatively new: there are population studies, hospital studies and primary healthcare studies. Most of these studies have analyzed the association between COPD and several isolated diseases; and a few of them have analyzed its association to larger groups of chronic diseases; however the strong differences between diseases from the two groups make their comparison difficult. COPD is a disease suffered by elder people and several associated diseases are frequent for their high prevalence among elder population; as a consequence a key point when studying comorbidity is to distinguish clearly if these diseases are independent or if they are associated more frequently than in general population [13].

The aim of this study was to quantify the prevalence of COPD, and quantify and describe diagnosed COPD-related chronic comorbidity in family practices in an area of Madrid, using EMR-based data.

## Methods

We conducted an observational, descriptive, cross-sectional study in a health area of the Madrid Autonomous Region (*Comunidad de Madrid*), based on a catchment population of 887,134 and data pertaining to 2007.

The study used the EMRs of 198,670 persons, corresponding to the practice population allocated to 129 FPs who, by way of inclusion criteria, met the following two EMR utilization quality requirements: 1) annotations in over 64% of medical visits (75^th^ percentile); and 2) a mean of more than four care episodes per patient.

Data were drawn from clinical histories. The following data were extracted from each patient: age; sex; and all diagnoses, grouped into Expanded Diagnosis Clusters (EDC), for which medical advice had been sought in 2007.

Patients were classified by means of the ACG® Case–Mix System, version 7, developed by Johns Hopkins University [14] on the premise that the level of resources needed for health care is related to disease burden. This system enables mutually exclusive groups of patients, Adjusted Clinical Groups (ACGs), to be formed on the basis of the criterion of similar use of resources, using age, sex and morbidity across a given period of time (usually one year) for the purpose. In addition, patients can be regrouped, according to their respective ACGs, into six Resource Utilisation Bands (RUBs), which are based on expected use and range from zero (RUB 0) to very high expected use (RUB 5). Furthermore, the system also generates categories, known as Expanded Diagnosis Clusters (EDCs), which group patients by reference to clinical criteria. The ACG ® Case – Mix System has been widely used and has proved its validity to study morbidity and comorbidity [15-18].

Using pre-established criteria [19], the research team made an initial selection of 40 chronic EDCs and, in a second stage, identified 26 so-called: “high prevalence–high impact” (HP/HI) [16] EDCs, which met one or both of the following criteria: 1) prevalence above the 50^th^ percentile; and/or 2) over 50% of patients included in RUBs 4 and 5 (elevated use of resources). We included COPD in this list of 26 EDCs, and analysed its association with the other 25 high prevalence - high impact chronic EDCs.

As a first step and using health records patients older than 40 and suffering EDC RES04 were selected. EDC RES04 includes emphysema, chronic bronchitis and COPD. Using this criterion for case definition, the prevalence of COPD seen in family practices was then calculated with its 95% confidence level (95% CI).

All statistical analyses were performed using the SPSS statistical package, version 15.0. In the case of qualitative variables, frequency distributions were calculated, along with their 95% CIs where pertinent; in the case of quantitative variables, means and their standard deviations were calculated, normality was tested, and parametric or non-parametric tests were applied, depending on the distribution of the variable.

Presence of comorbidity was analysed, by quantifying the proportion of patients included in each of the designated 6 comorbidity levels, ranging from isolated COPD to COPD associated with 5 or more comorbidities. The association between COPD and each of the 25 HP/HI diseases studied was quantified, and standardised prevalence ratios (observed/expected prevalence) were calculated, assuming independence between the diseases analysed and COPD. For the calculation of expected frequencies, age and sex were adjusted for using the indirect method.

The study was approved by the Ethics Committee of the Puerta de Hierro University Teaching Hospital.

## Results

The 129 FPs covered by the study attended a total practice population of 198,670 patients, made up of 104,003 women (52.35%) and 94,667 men (47.65%). Of the 3,183 patients diagnosed with COPD, 3,124 (98%) were aged 40 years or over, comprising 2,376 (76%) men and 748 (24%) women. Patients’ mean age was 71.41 ± 11.50 years, without significant differences between the sexes. COPD prevalence in the family practices targeted was 3.2% (95% CI 3.0 – 3.3), and was higher in men than in women (5.3% vs. 1.4%), a difference of 3.9%, p < 0.01.

COPD patients are a population with an elevated comorbidity. They have a mean of 4 ± 2.04 chronic HP/HI diseases. The number of diseases was higher in women than in men (Mann–Whitney U-test, *p* < 0.01) and rose with age (Table 1). Only 9% of patients had isolated COPD, and over half had three or more chronic problems in addition to COPD (Table 2). Table 3 shows the percentage distribution of the 25 chronic diseases studied among COPD patients, overall and by sex: 52% of patients presented with arterial hypertension, 34% with lipid metabolism disorders, 25% with obesity, 20% with diabetes, 15% with arrhythmia and 14% with thyroid disease. Men had more ischaemic heart disease, generalised artherosclerosis, diabetes mellitus, chronic liver disease, malignant neoplasms and chronic renal failure (*p* < 0.01). Women suffered more from asthma, arterial hypertension, osteoporosis, thyroid disease, degenerative joint disease and anxiety/depression (*p* < 0.01).

**Table 1:** Chronic diseases / Patient

**Age (years)**	**Men**	**Women**	**Total**
40 – 49	2.47 ± 1.44	2.42 ± 1.25	2.45 ± 1.37
50 – 59	2.88 ± 1.57	3.23 ± 1.52	2.96 ± 1.56
60 – 69	3.41 ± 1.82	3.94 ± 1.80	3.52 ± 1.83
70 – 79	4.25 ± 2.11	4.50 ± 1.96	4.30 ± 2.09
80 and over	4.55 ± 2.06	4.76 ± 1.96	4.61 ± 2.03
**Total**	**3.93 ± 2.06**	**4.17 ± 1.97**	**3.99 ± 2.04**

**Table 2:** Comorbidity levels in patients with COPD

**Comorbidity**	**Men**	**Women**	**Total**
	**N (%)**	**N (%)**	**N (%)**
COPD alone	242 (10.19)	46 (6.15)	288 (9.22)
COPD + 1 or more	2134 (89.81)	702 (93.85)	2836 (90.78)
COPD + 2 or more	1706 (71.80)	593 (79.28)	2299 (73.59)
COPD + 3 or more	1262 (53.11)	452 (60.43)	1714 (54.87)
COPD + 4 or more	829 (34.89)	293 (39.17)	1122 (35.92)
COPD + 5 or more	505 (21.25)	164 (21.93)	669 (21.41)

**Table 3:** Prevalence of chronic diseases in patients with COPD

**EDC**	**Prevalence Men**	**Prevalence Women**	**Prevalence Total**
**Arterial hypertension**	49.96	58.42	**51.98**
**Disorders of lipid metabolism**	34.22	35.16	**34.44**
**Obesity**	24.66	27.41	**25.32**
**Diabetes mellitus**	22.26	16.04	**20.77**
**Anxiety/Depression**	16.58	31.68	**20.20**
**Cardiac arrhythmia**	15.99	15.24	**15.81**
**Thyroid disease**	10.94	24.60	**14.21**
**Malignant neoplasms**	14.56	7.62	**12.90**
**Generalised atherosclerosis**	12.46	4.14	**10.47**
**Ischaemic heart disease**	9.30	5.75	**8.45**
**Deafness, hearing loss**	7.74	9.63	**8.19**
**Congestive heart failure**	7.70	9.09	**8.03**
**Cerebrovascular disease**	7.91	6.15	**7.49**
**Osteoporosis**	2.65	20.72	**6.98**
**Chronic renal failure**	7.11	3.88	**6.34**
**Asthma**	3.75	12.43	**5.83**
**Degenerative joint disease**	4.08	10.70	**5.67**
**Glaucoma**	4.97	6.42	**5.31**
**Chronic liver disease**	5.72	2.67	**4.99**
**Chronic skin ulcer**	2.36	2.41	**2.37**
**Cardiac valve disease**	2.31	1.87	**2.21**
**Dementias**	1.85	2.14	**1.92**
**Parkinson’s disease**	1.73	1.47	**1.66**
**Schizophrenia**	1.09	1.34	**1.15**
**Benign prostatic hypertrophy**	20.71		

EDC: expanded diagnosis cluster.

To control for the effect of age, Table 4 shows observed and expected prevalences adjusted for age and sex. In COPD patients, ten of the 25 diseases studied were more frequent than would have been expected, and the following were particularly noteworthy, with congestive heart failure being 2.4 times, chronic liver disease 89%, asthma 73%, generalised artherosclerosis 67%, osteoporosis 42% and ischaemic heart disease 41% more frequent. The remaining fifteen studied diseases present no meaningful differences in prevalence with respect to those found in general population even though some of them are very frequent among patients suffering COPD.

**Table 4:** Standardised prevalence of chronic diseases in patients with COPD

**EDC**	**Observed prevalence**	**Expected prevalence**	SPR^*^	**Lower CI**	**Upper CI**
	**% COPD**	**% COPD**			
**Congestive heart failure**	8.03	3.34	**2.41**	1.88	2.93
**Chronic liver disease**	4.99	2.63	**1.89**	1.37	2.42
**Asthma**	5.83	3.36	**1.73**	1.29	2.18
**Generalised atherosclerosis**	10.47	6.25	**1.67**	1.35	1.99
**Osteoporosis**	6.98	4.91	**1.42**	1.09	1.75
**Ischaemic heart disease**	8.45	6.00	**1.41**	1.11	1.71
**Schizophrenia**	1.15	0.85	**1.35**	0.57	2.12
**Thyroid disease**	14.21	11.06	**1.28**	1.07	1.50
**Chronic renal failure**	6.34	4.96	**1.28**	0.96	1.59
**Anxiety/Depression**	20.20	15.86	**1.27**	1.10	1.45
**Cardiac valve disease**	2.21	1.76	**1.25**	0.73	1.77
**Cardiac arrhythmia**	15.81	12.70	**1.25**	1.05	1.44
**Obesity**	25.32	20.49	**1.24**	1.08	1.39
**Chronic skin ulcer**	2.37	1.96	**1.20**	0.72	1.69
**Glaucoma**	5.31	4.51	**1.18**	0.86	1.46
**Cerebrovascular disease**	7.49	6.48	**1.16**	0.89	1.42
**Diabetes mellitus**	20.77	18.24	**1.14**	0.98	1.29
**Degenerative joint disease**	5.67	5.11	**1.11**	0.82	1.40
**Malignant neoplasms**	12.90	11.63	**1.11**	0.92	1.30
**Disorders of lipid metabolism**	34.44	31.32	**1.10**	0.98	1.22
**Arterial hypertension**	51.98	48.14	**1.08**	0.99	1.17
**Deafness, hearing loss**	8.19	7.87	**1.04**	0.82	1.27
**Parkinson’s disease**	1.66	1.71	**0.97**	0.50	1.44
**Benign prostatic hypertrophy**	15.81	16.41	**0.96**	0.81	1.11
**Dementias**	1.92	2.56	**0.75**	0.41	1.08

EDC: expanded diagnosis cluster.

(*) Standardised prevalence ratio.

## Discussion

COPD is a disease that affects 3.2% of the population attended by FPs; 90% of patients had one or more related diseases and 55% presented with three or more additional chronic diseases.

The morbidity seen in primary health care comes close to that reported in population-based studies. In a health care system such as Spain’s, citizens are not only required to register with a FP, but, in order to be eligible for hospital care, must first consult their FP.

In recently published population-based epidemiological study conducted in Spain, COPD prevalence was estimated at 10.2%. It is well known that COPD tends to be underdiagnosed by FPs [20-22]. In our case a prevalence of 3.2% falls in the range found by Halbert [2] in the studies that used respiratory symptoms based medical diagnosis as case definition. Comparing to the prevalence data from the previously referenced study [3] which used GOLD criteria as case definition, family doctors might have been underdiagnosing 69% of the patient’s population suffering COPD. This is a high underdiagnosis ratio, comparable with that of previous studies, and it has scarcely changed in the last ten years.

At the date of our study, all health centres were supplied with spirometers but FPs are widely known to make little use of this particular diagnostic technique [23-25]. Furthermore, underdiagnosis may also be influenced by FPs’ attitude to this disease: indeed, reluctance to label the patient is such that treatment may even be initiated before the formal diagnosis has been entered on the clinical record [26].

Of the total, only 9% of patients presented with COPD alone, and the remainder tended to have a mean of four of the 25 chronic diseases studied. Being an aged population, the diseases or conditions involved are not only specific to older persons, but are also present at a frequency that is higher than expected, e.g., heart failure, arrhythmia, ischaemic heart disease, anxiety/depression, obesity, thyroid disease, asthma, osteoporosis, generalised artherosclerosis and chronic liver disease. Smoking-related inflammation is postulated as a possible cause of many of these diseases [7].

While other studies have addressed the problem of comorbidity in patients with COPD, most have focused on a few isolated diseases. We, in contrast, studied the association between COPD and a further 25 chronic diseases. We found three studies which analysed the association between COPD and a long list of diseases in primary care patients but, as in the case of other comorbidity studies, comparison was hindered by methodological differences, in that two were based on data from ongoing records kept by volunteer physicians [27,28] and the third used data drawn from EMRs [29]. All three used the same case definition as ours, namely, COPD recorded by a FP. Results were controlled for age in only one study [27]. Although the disease list used was not the same throughout, there was a substantial core group of diseases that was common to all the studies, including ours. As in our case, the other three studies also observed a high prevalence of cardiovascular diseases and osteoporosis.

COPD patients who also present with cardiovascular diseases register more episodes of COPD exacerbation, more hospital admissions, a notable increase in cost of medical care [30] and higher cardiovascular disease mortality [31]. In our case, 16% of patients presented with arrhythmia, 8.5% with ischaemic heart disease, and a further 8% with heart failure. This association has been reported in other studies [32]. In a study focusing on family practices in Spain, de Miguel found that prevalence of heart disease among COPD patients was 19% [8].

As reported by most studies [8,32-34], cardiovascular risk factors, such as arterial hypertension, lipid metabolism disorders, diabetes and obesity, are among the most prevalent diseases in COPD patients. This, however, is due to the ageing of patients, since, once age had been controlled for, prevalence did not prove very different to what might have been expected (Table 4). This finding indicates that, when studying the association between COPD and cardiovascular diseases, the confounding role of risk factors must be taken into account [33].

We observed 89% more chronic liver diseases than expected in a general population of this age and sex. This association has been little described but a Japanese-based study, targeting family medicine offices attended by specialist physicians [35], reported an elevated prevalence of chronic liver disease, chronic hepatitis C, hepatic steatosis and alcoholic hepatitis. Another study [36] similarly observed this association between liver disease and COPD, after controlling for age, sex and smoking habit. In addition, COPD has been described as having a swifter progression in hepatitis C patients [37]. Although, the aetiopathogenic mechanisms of this association are unclear, an interleukin-8 mediated chronic inflammatory process is suspected as being the cause [38].

Furthermore, 7% of COPD patients have osteoporosis, with a frequency that is 10 times greater in women than in men, and an observed prevalence that is higher than expected for patients of their age and sex. Some studies report patients with COPD as having a threefold risk of presenting with osteoporosis [19,21]. Since a number of studies have shown that inhaled corticoids have no effect on bone density, other factors, such as reduced mobility, smoking habit or adverse effects of other drugs, may be the cause of this.

Anxiety and depression are frequent among patients with COPD. In our study, 20% of patients presented with these problems, with a frequency that was twice as high in women as in men. In one review, prevalence varied widely between some studies and others, due in part to the measurement instrument used and type of patient studied. Among stable patients, prevalence of depression ranged from 10% to 42% and prevalence of anxiety from 10% to 19% [39]. Our data are in line with those published. The causes for the increase in affective disorders in these patients are uncertain but, in addition to the reactive effect due to deterioration in the state of health, nicotine dependence may play an important role [40].

Six per cent of our patients had COPD and asthma, with a frequency that was more than three times higher in women than in men. This prevalence is lower than that observed in population-based studies [41], a finding probably explained by the same type of underdiagnosis as that which affects COPD. This overlapping of diseases in the same patient represents a diagnostic and therapeutic challenge for FPs [42,43].

This study suffers from some limitations: firstly, there are those inherent in any retrospective design which uses EMRs as its data source. As a data source, medical records introduce biases stemming from under-registration and the poor quality of the data recorded [44]. In an attempt to solve this problem, physicians were selected according to the quality of their records.

Another limitation derives from the case-definition criterion used, i.e., the need for patients’ clinical histories to show evidence of diagnosis of COPD, since FPs are well known for making little use of spirometry and for misclassifying COPD patients [45]. However, in view of the fact that this was a chronic disease and of the longitudinal nature of primary care, it is likely that underdiagnosis involved the milder forms of COPD [22,46] and that misclassification was due to COPD being diagnosed as asthma and vice-versa.

## Conclusions

COPD is pathology with high sanitary, social and economic impact. Patients suffering COPD present high co morbidity associated to other pathologies of high impact on healthcare services; as a consequence COPD patients stratification should regard the comorbidity burden in addition to the degree of obstruction.

The clinical practice guidelines that are in place are targeted at isolated diseases and do not envisage the existence of comorbidity. Moreover, the healthcare delivery models based on diseases management offer a poor response to comorbidity problem and tend to fragment the assistance to the patient inciding negatively on healthcare services [47]. In addition, the emerging healthcare model based on telemonitoring and treatment “is going home” [48], and providers of such care need to customise services involved in following up and monitoring chronically ill patients in their homes. Consequently, comorbidity poses a challenge for the care of COPD patients, and so studies are needed that will help specify these different patient profiles.

In view of the results of this study, there is a need for improving the diagnosis of COPD in family practices, there is a need for clinical practice guidelines to be reviewed and rendered patient- rather than disease-oriented [49]. Similarly there is a need to adapt patient telemonitoring programmes and applications [48], as well as care-delivery models in general, so that these can adopt an integrated approach patient-centered, based on longitudinal care and co-ordination.

### Ethics approval

All patient registration data were treated confidentially pursuant to the Spanish 1999 Personal Data Protection Act (*Ley Orgánica de Protección de Data de Carácter Personal*). The data were organised in a database and made available for analysis in this project and for research in future projects.

Patients were registered by age and sex only. No electronic patient identifier was used, and there was no information that could be used to identify individual registry patients by personalised analysis of the data. Participants’ identity was deemed confidential.

## Abbreviations

ACG: Adjusted clinical groups; CI: Confidence interval; COPD: Chronic obstructive pulmonary disease; EDC: Expanded diagnosis clusters; EMR: Electronic medical record; FP: Family physician; GOLD: Global strategy for diagnosis, management, and prevention of COPD; HP/HI: High prevalence/ high impact; RUB: Resource utilisation bands.

## Competing interests

L. García-Olmos, A. Alberquilla, P. García-Sagredo, L. Morales, M. Carmona, M.J. de Tena-Dávila, M. Pascual, A. Muñoz, C.H. Salvador and J.L. Monteagudo declare that they have no conflicts of interest.

V. Ayala is an employee of Praxair España.

## Authors’ contributions

LG-O, AA and VA contributed to the study design. CHS and LG-O conceived the study. AA, PG-S, MC and CHS supervised the study. AA, MP and AM assessed procedures and built a database. AA, PG-S, MC and MJdT-D analysed the data. LG-O, VA and LM wrote the first and last drafts of the manuscript. All authors contributed ideas, revised different drafts of the manuscript and approved the final version.

## Pre-publication history

The pre-publication history for this paper can be accessed here:

http://www.biomedcentral.com/1471-2296/14/11/prepub
